# Biplane Enhancement Coil for Magnetic Induction Tomography of Cerebral Hemorrhage

**DOI:** 10.3390/bios14050217

**Published:** 2024-04-26

**Authors:** Zhongkai Cao, Bo Ye, Honggui Cao, Yangkun Zou, Zhizhen Zhu, Hongbin Xing

**Affiliations:** 1 School of Information Engineering and Automation, Kunming University of Science and Technology, Kunming 650500, China; 20212104051@stu.kust.edu.cn (Z.C.); 20221104006@stu.kust.edu.cn (H.C.); xinghongbin@stu.kust.edu.cn (H.X.); 2 Yunnan Key Laboratory of Intelligent Control and Application, Kunming 650500, China; 3 College of Civil Aviation and Aeronautics, Kunming University of Science and Technology, Kunming 650500, China; zouyangkun@kust.edu.cn; 4 The First Military Representative Office of the Chongqing Military Representative Bureau of the Army Equipment Department in Kunming, Kunming 650000, China; zhizhen99666@126.com

**Keywords:** magnetic induction tomography, cerebral hemorrhage, coil design, target field method, magnetic field linearity

## Abstract

Magnetic Induction Tomography (MIT) is a non-invasive imaging technique used for dynamic monitoring and early screening of cerebral hemorrhage. Currently, there is a significant challenge in cerebral hemorrhage MIT due to weak detection signals, which seriously affects the accuracy of the detection results. To address this issue, a dual-plane enhanced coil was proposed by combining the target field method with consideration of the spatial magnetic field attenuation pattern within the imaging target region. Simulated detection models were constructed using the proposed coil and cylindrical coil as excitation coils, respectively, and simulation imaging tests were conducted using the detection results. The simulation results indicate that compared to the cylindrical coil, the proposed coil enhances the linearity of the magnetic field within the imaging target region by 60.43%. Additionally, it effectively enhances the detection voltage and phase values. The simulation results of hemorrhage detection show that the proposed coil improves the accuracy of hemorrhage detection by 18.26%. It provides more precise detection results, offering a more reliable solution for cerebral hemorrhage localization and detection.

## 1. Introduction

**Figure 1:**
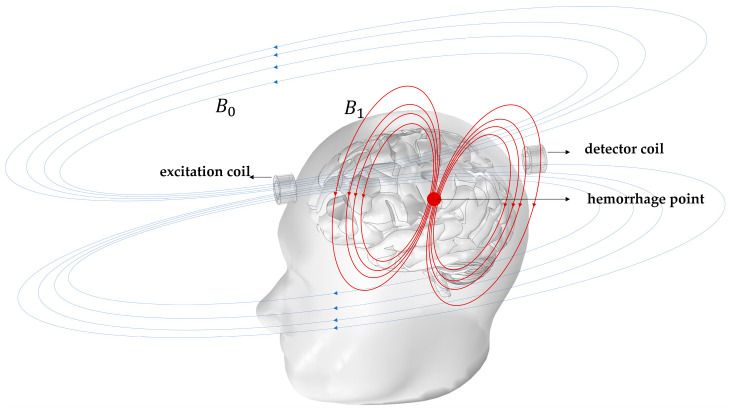
Principles of Magnetic Induction Tomography detection of cerebral hemorrhage.

Cerebral hemorrhage (CH) is a type of cerebrovascular disease. It can cause bleeding or blood clots to compress cerebral nerves, potentially leading to cerebral infarction. CH is the leading cause of death in China; early and real-time dynamic monitoring can significantly lower the risk of mortality [1,2,3]. Currently, the mainstream cerebral hemorrhage imaging technologies include X-ray, CT, MRI, etc. [4,5], which have mature technologies and high imaging accuracy. These technologies and equipment often come with a high cost. Furthermore, some of them utilize radiation sources that pose potential risks to human health, rendering them unsuitable for prolonged dynamic monitoring [6]. Magnetic Induction Tomography (MIT) is a non-contact tomographic scanning technique. It reconstructs images by utilizing alternating magnetic fields and the passive electromagnetic properties of the object under examination [7]. The basic principle of MIT imaging of cerebral hemorrhage is shown in Figure 1. The excitation coil generates a primary magnetic field in the imaging target area, and this alternating magnetic field generates an induced voltage on the detection coil. When a hemorrhage exists inside the imaging area, a secondary magnetic field $B_{1}$ is generated at the hemorrhage location due to the alternating magnetic field $B_{0}$, and the induced voltage will be disturbed [8]. This change can be characterized as phase information contained in the change in the value of the induced voltage across the detection coil [9]. When the excitation coil is positioned at a fixed location, a set of measurements for $B_{1}$ can be obtained by the detection coils located around the imaging target area at other positions. Afterwards, by rotating the excitation coil along the cross-sectional circumference, multiple sets of $B_{1}$ measurement values can be obtained. Reconstructing the obtained multiple sets of measured values using a certain image reconstruction algorithm, the resulting image can reflect the distribution of conductivity of different tissues within the imaging target area on the cross-section [10,11,12,13].

Research on cerebral hemorrhage MIT technology primarily focuses on several aspects, including brain model construction, hardware system development, and imaging algorithm enhancement. These studies aim to improve the imaging performance of cerebral hemorrhage MIT technology, enabling more accurate detection and localization of cerebral hemorrhage lesions. In response to the improvement of the MIT hardware system for cerebral hemorrhage, Al-zeibak et al. first proposed the application of MIT technology to biological tissue detection in 1993. They designed a dual-coil MIT system to acquire annular detection signals through mechanical rotation. However, the mechanical rotation introduced considerable noise interference, resulting in limited data acquisition [14]. Korjenevsky et al. developed a multi-channel MIT system for biological tissue detection. It consisted of 16 excitation and detection coils, capable of reconstructing the conductivity distribution of the human brain at an excitation frequency of 20 MHz [15]. Z. Zhang et al. replaced the excitation coil in the excitation-receiver coil sensor array from a cylindrical coil to a two-armed Archimedean helical coil (TAASC) to obtain an improved coil system, which effectively increased the phase detection sensitivity of the sensor array [16]. Merwa et al. constructed a simulation model consisting of 16 excitation coils and 32 detection coils. By combining excitation and detection coils, they obtained 16 planar gradient coils (PGRAD). Through simulation experiments, they validated the effectiveness of PGRAD in enhancing imaging spatial resolution [17]. Soleimani designed an eight-coil rotation system based on the Rotation Matrix Imaging Tomography (RMIT). The coil array rotates around the central axis perpendicular to the plane of the coil array to increase the number of independent measurement data, thereby generating higher-quality images. However, the rotation process requires manual operation [18]. Li Ke et al. developed a fan MIT system to realize the reconstruction of the conductivity distribution in the imaging target area by means of fan-beam scanning [19]. Chenyang Wang utilized a dual-figure-eight coil configuration to construct a deep brain tissue MIT system. Test results demonstrate that the dual-figure-eight coils effectively enhance the sensitivity of phase signals in detecting deep brain tissues, thereby providing a solid data foundation for subsequent data processing and imaging. This offers new research insights for deep brain tissue MIT detection [20]. Hongbo Qi proposed a sensor array with dual Helmholtz coils as excitation coils to provide a uniform magnetic field for the imaging target region. This approach increases the detection phase offset and enhances the accuracy of deep brain hemorrhage detection [21]. S. Haikka et al. proposed a helmet-like coil array consisting of 31 circular coils, which can effectively improve the imaging quality at 10 MHz frequency [22].

Significant progress has been made in the research on hardware systems for cerebral hemorrhage MIT. However, there are still challenges related to weak detection voltage and phase signals. This may lead to signal offset due to potential external interference, poor magnetic field linearity, or system errors, resulting in inaccurate imaging results. Furthermore, weak detection signals pose high demands on signal processing techniques, such as signal enhancement, filtering, and noise suppression, thereby increasing the complexity of data processing. One of the key reasons for the weak detection signals is the presence of fluctuation drifts in the magnetic field within the imaging region. Therefore, precise control of its distribution is necessary to reduce interference with the detection signals. In this paper, a dual-plane enhanced excitation coil for cerebral hemorrhage MIT systems is proposed using the target field method. It enhances the magnetic field intensity within the imaging target region through magnetic coupling effects. Additionally, the design considers the attenuation pattern of the spatial magnetic field to more accurately control the distribution of magnetic fields at various locations within the imaging target region. Through simulation analysis, the designed excitation coil effectively enhances the strength of the detected voltage and phase signals. Moreover, the magnetic field generated within the imaging target area exhibits better linearity, thereby improving the system’s dynamic range and detection reliability. The Section 2 elaborates on the specific methods and procedures for coil design, while the Section 3 constructs the coil model and conducts simulation analysis. The conclusion is given at the end.

## 2. Design Methodology

**Figure 2:**
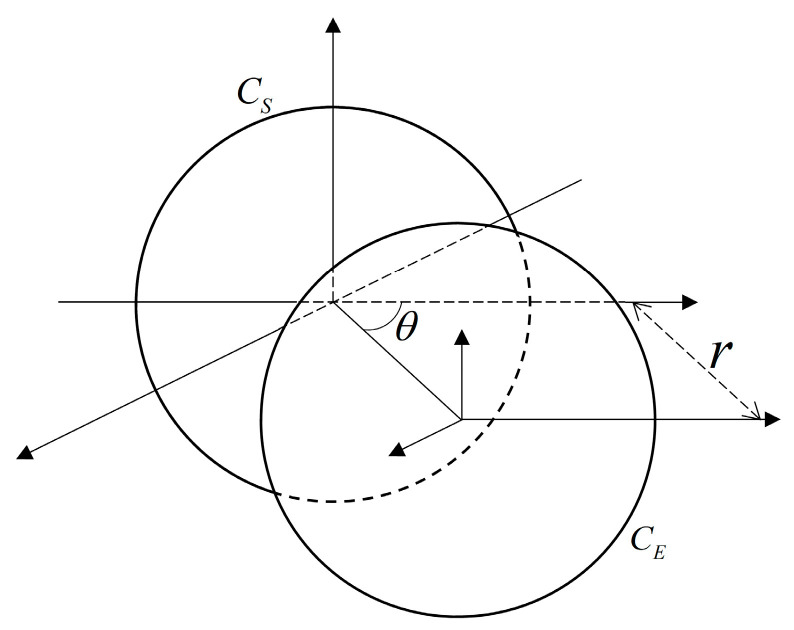
The relative positional relationship between the planar coils $C_{S}$ and $C_{E}$ comprising the double-planar enhanced coil.

The relative position of the biplane enhancement coil is shown in Figure 2. On the basis of the existing excitation coil CE, an enhancement coil CS is added to form a dual-plane enhanced excitation coil, achieving enhancement of the magnetic field intensity within the imaging target region.

According to the magnetic coupling theory, the magnetic coupling coefficient *k* between two coils can be expressed as follows:

(1) $$k=\frac{\phi_{SE}}{I_{E}}=\frac{\phi_{ES}}{I_{S}}$$

where $\phi_{SE}$ and $\phi_{ES}$ are the magnetic flux generated on coils $C_{S}$ and $C_{E}$ by coils $C_{E}$ and $C_{S}$ with currents $I_{E}$ and $I_{S}$, respectively. The value of *k* indicates the degree of magnetic coupling between the two coils, and its magnitude is related to the angle between the two coils, the shape of the coil, and other factors. When θ is 90°, the mutual coupling area of the two coils is the largest, and the magnetic coupling effect is the strongest, which can effectively enhance the magnetic field strength in the imaging target area. After determining the relationship between the relative positions of the two coils, the design of its wire arrangement is then deduced. 

**Figure 3:**
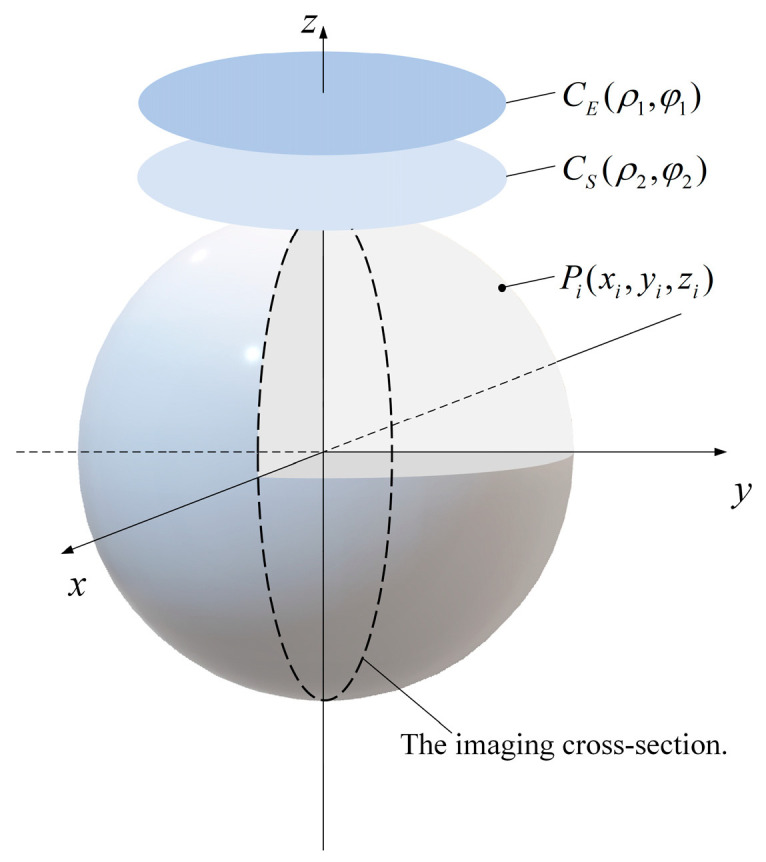
Location of biplane enhancement coil in relation to the target imaging region.

The target field method is a mathematical approach used for designing electromagnetic coils. Its basic idea is to predefine the desired electromagnetic field distribution as a specific target. Then, through inverse deduction, the current distribution or properties and parameters of the magnetic field source that will produce this target field are determined [23]. This method enables precise electromagnetic field control. By applying this method to design coils, ideal coil shapes and parameters can be obtained. The specific relationship between the plane where the coil is located and the imaging target area is shown in Figure 3.

**Figure 4:**
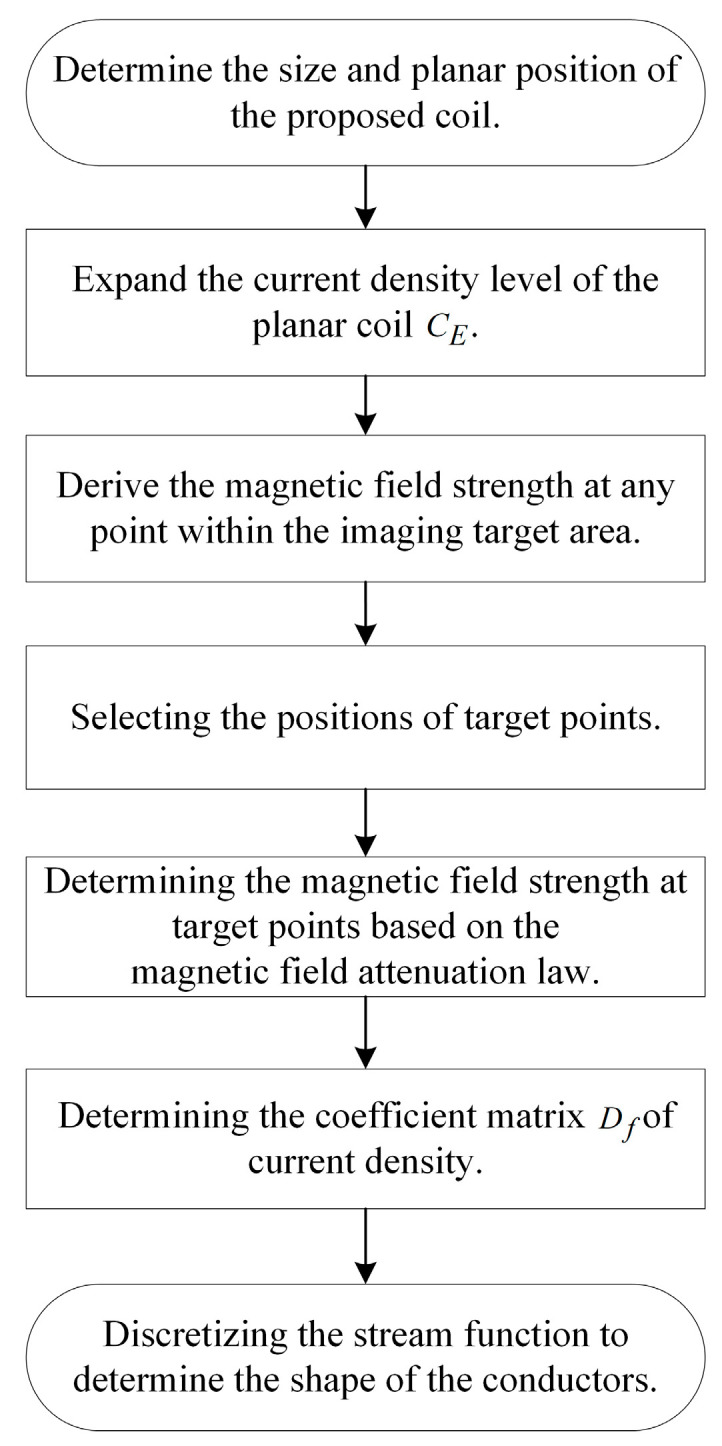
Flowchart for coil design.

Since the electromagnetic field distribution within the imaging target area is predefined, the distance between $C_{S}$ and $C_{E}$, as well as their distances from the imaging target area, only affect the shape of the coil and not the magnetic field distribution. Here, the distance is taken as 2 mm, and $P_{i}$ indicates the target point in the imaging target area where the desired magnetic field strength is set. The plane represented by the dashed lines in the figure is the imaging cross-section that needs to be imaged. The coil design flowchart is shown in Figure 4.

After determining the location of the coil plane, the current density in the plane where the excitation coil is located is expanded in steps.

(2) $$\{\begin{array}{c} J_{\rho_{i}}=\sum_{f}^{F}D_{f}\frac{k}{\rho_{i}}\cdot \sin[fc(\rho_{i}-\rho_{0})]\sin k\rho_{i},i=1,2,\ldots \\ J_{\varphi_{i}}=\sum_{f}^{F}D_{f}fc\cdot \cos[fc(\rho_{i}-\rho_{0})]\cos k\varphi_{i},i=1,2,\ldots \end{array}$$

where $J_{\rho_{i}}$ and $J_{\varphi_{i}}$ are the radial and tangential current densities in the plane where $C_{S}$ and $C_{E}$ are located, respectively. $D_{f}$ is the matrix of current density expansion coefficients to be solved, *F* is the number of target points to be selected, *k* is a constant, and *c* is calculated as follows:

(3) $$c=\frac{\pi }{\rho_{m}-\rho_{0}}$$

where, when $C_{S}\;$ and $C_{E}\;$ are of the same size, $\rho_{m}$ and $\rho_{0},$ respectively represent the maximum and minimum radii of the conductors arranged on the plane. According to the Biot-Savart law, the magnetic field strength excited at any point in the imaging target area by a single planar coil oriented perpendicular to the coil plane can be determined.

(4) $$B=\frac{\mu_{0}}{4}\int_{\rho_{0}}^{\rho_{m}}\int_{0}^{2\pi }\frac{\rho d\rho d\varphi }{R^{2}}[\left(J_{\rho }\cos\varphi -J_{\varphi }\sin\varphi \right)(y-\rho \sin\rho )-\left(J_{\rho }\sin\varphi +J_{\varphi }\cos\varphi \right)(x-\rho \cos)]$$

where *R* is the distance between any point in the imaging target area and the excited magnetic field current element in the coil plane. As the proposed coil consists of two planar coils, the magnetic field strength at any point within the imaging target area should be the superposition of the magnetic field strengths generated by the two coils:

(5) $$\begin{array}{l} B=\frac{\mu_{0}}{4}\int_{\rho_{0}}^{\rho_{m1}}\int_{0}^{2\pi }\frac{\rho_{1}d\rho_{1}d\varphi_{1}}{{R_{C_{E}}}^{3}}[\left(J_{\rho_{1}}\cos\varphi_{1}-J_{\varphi_{1}}\sin\varphi_{1}\right)\left(y_{i}-\rho_{1}\sin\rho_{1}\right)-\left(J_{\rho_{1}}\sin\varphi_{1}+J_{\varphi_{1}}\cos\varphi_{1}\right)\left(x_{i}-\rho_{1}\cos\varphi_{1}\right)]\\ +\frac{\mu_{0}}{4}\int_{\rho_{0}}^{\rho_{m2}}\int_{0}^{2\pi }\frac{\rho_{2}d\rho_{2}d\varphi_{2}}{{R_{C_{S}}}^{3}}[\left(J_{\rho_{2}}\cos\varphi_{2}-J_{\varphi_{2}}\sin\varphi_{2}\right)\left(y_{i}-\rho_{2}\sin\rho_{2}\right)-\left(J_{\rho_{2}}\sin\varphi_{2}+J_{\varphi_{2}}\cos\varphi_{2}\right)\left(x_{i}-\rho_{2}\cos\varphi_{2}\right)] \end{array}$$

Bring Equation (2) into Equation (5) and simplify:

(6) $$B=\sum_{f=1}^{F}G_{f}D_{f}$$

Each element in ***B*** represents the magnetic field strength value at any point within the imaging target area. $G_{f}$ is a function about an arbitrary point $P_{j}(x_{j},y_{j},z_{j})$ within the imaging target region, so that the current density coefficient matrix $D_{f}$ can be solved by solving Equation (6) in matrix form Equation (7):

(7) $$\left[\begin{array}{c} B_{1}\\ \begin{array}{c} B_{2}\\ B_{3}\\ \begin{array}{c} \vdots \\ B_{F} \end{array} \end{array} \end{array}\right]=\left[\begin{array}{cc} \begin{array}{ccc} G_{11} & G_{12} & G_{13}\\ G_{21} & G_{22} & G_{23}\\ G_{31} & G_{32} & G_{33} \end{array} & \begin{array}{cc} \cdots & G_{1F}\\ \cdots & G_{2F}\\ \cdots & G_{3F} \end{array}\\ \begin{array}{ccc} \vdots & \vdots & \vdots \\ G_{F1} & G_{F2} & G_{F3} \end{array} & \begin{array}{cc} \ddots & \vdots \\ \cdots & G_{FF} \end{array} \end{array}\right]\left[\begin{array}{c} D_{1}\\ D_{2}\\ D_{3}\\ \vdots \\ D_{F} \end{array}\right]$$

To better optimize the coil design and achieve the desired magnetic field distribution, as well as enhance the stability of the magnetic field and reduce unnecessary fluctuations, consideration is given to the attenuation law of the magnetic field in space when determining the target points and their corresponding magnetic field strength. Typically, this law can be described using an attenuation model:

(8) $$B\left(r\right)=\frac{B_{0}}{\sqrt{1+{\left(\frac{r}{\lambda }\right)}^{2}}}$$

where $B_{0}$ represents the magnetic field strength at the start point, B(r) is the magnetic field strength at a distance *r* from the start point, and λ is the magnetic field decay length. Assuming that the magnetic field strength in the imaging target area of uniform attenuation, at this time λ is a constant. When the value is determined, the above equation can be introduced from the starting point at different distances from the target point corresponding to the value of the magnetic field strength. Square both sides of the above equation and multiply by $1+{\left(\frac{r}{\lambda }\right)}^{2}$, after finishing:

(9) $$\lambda =\frac{rB\left(r\right)}{\sqrt{B_{0}^{2}-B{\left(r\right)}^{2}}}$$

From the equation above, it is understood that given the distance r from a starting point and the magnetic field strength B(r) at that point, the magnetic field decay length L under corresponding conditions can be determined. The imaging target area was set as a spherical region with a radius of 85 mm, based on the typical head circumference of an adult. A cylindrical excitation coil was positioned at the coil location indicated in Figure 3, and measurements of the magnetic field strength at various positions within the imaging cross-section of the imaging target area under ideal conditions were conducted. The obtained results were then substituted into Equation (9) for the calculation of λ. The final decay model obtained is represented by Equation (11). The purpose of adding a constant in the denominator is to signify that, at locations closer to the excitation coil, the rate of magnetic field attenuation is lower, indicating a slower initial decay. Subsequently, the decay accelerates at a faster rate. The final attenuation index model is shown in Equation (10).

(10) $$B\left(r\right)=\frac{B_{0}}{\sqrt{1+{\left(\frac{0.5r}{1.2462}\right)}^{2}}+0.1}$$

**Figure 5:**
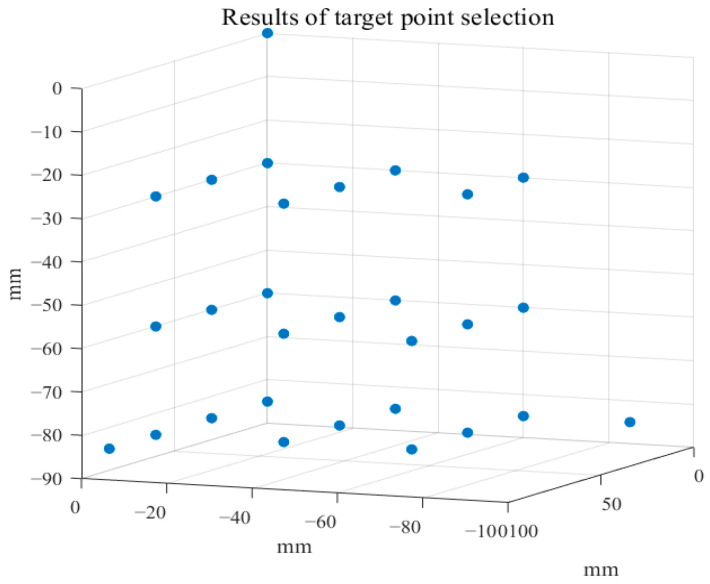
Target point selection results in the imaging area.

Next, the selection of target points is conducted. To reduce the ill-posedness in the design process, target points are chosen within the first quadrant of the imaging area. The final selected positions of the target points $P_{i}$ are shown in Figure 5, with a total of 29 points selected. The magnetic field strength at each point is determined by the obtained attenuation index model. By substituting these values into Equation (7), $D_{f}$ can be obtained.

In order to discretize the current density distribution, the stream function is introduced. By discretizing the current density, the continuous current distribution can be transformed into discrete current elements. These elements can then be arranged in space according to certain patterns, thereby forming the layout path of the coil conductors [24]. It is defined as follows:

(11) $$\psi \left(\rho ,\varphi \right)=\int_{0}^{\rho }\int_{0}^{2\pi }\left(\frac{\partial J_{\varphi }}{\partial \rho }-\frac{\partial J_{\rho }}{\partial \varphi }\right)d\rho d\varphi$$

Its discretization enables the following equation to be obtained:

(12) $$\psi \left(\rho ,\varphi \right)=-\overset{F}{\underset{f=1}{\sum }}D_{f}sin\left[fc\left(\rho -\rho_{0}\right)\right]cos\varphi$$

The points on the same contour of the function indicate the same magnitude of the current, and these contours are the paths of the energized wires. When the number of turns of the planar coil is *N*, the *N* contours corresponding to Equation (12) can be expressed as follows:

(13) $$\psi {\left(\rho ,\varphi \right)}^{\prime }=\psi {\left(\rho ,\varphi \right)}_{min}+(m+\frac{1}{2})I_{0}$$

where m=1,2,⋯N−1 and

(14) $$I_{0}=\frac{\psi {\left(\rho ,\varphi \right)}_{max}-\psi {\left(\rho ,\varphi \right)}_{min}}{N}$$

**Figure 6:**
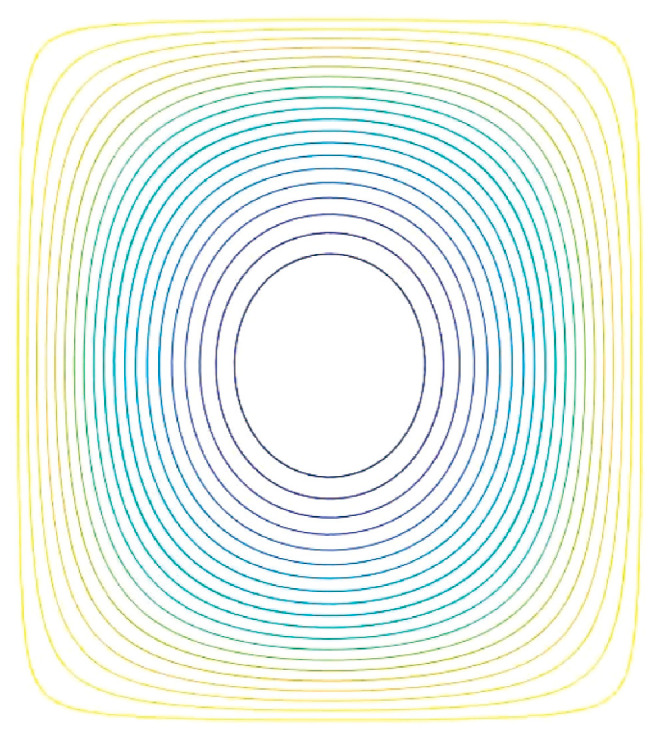
The winding pattern of the individual planar coil conductor in the final design.

${\Psi (\rho ,\varphi )}_{\max}$ and ${\Psi (\rho ,\varphi )}_{\min}$ are the maximum and minimum values of ${\Psi (\rho ,\varphi )}^{\prime }$, respectively. The maximum radius of the designed coil is 3 cm, and the minimum radius is 1 cm. The number of turns of the coil is set to 20 turns. $I_{0}$ represents the current value of the coil. The final calculation yields $I_{0}=1.0087A$. Once $I_{0}$ is determined, the equipotential lines of the stream function at $I_{0}$ represent the winding pattern of the coil. This is illustrated in Figure 6.

**Figure 7:**
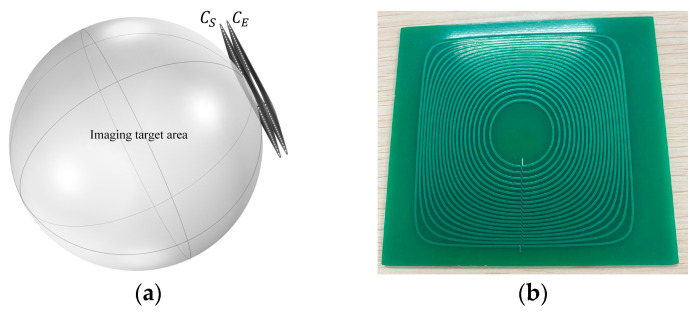
Final design results: (**a**) The relative positioning between the constructed coil simulation model and the imaging target area.; (**b**) The photograph of the proposed coil.

Since the positions and current densities of the two coil planes have been determined during the design process, the two plane coils comprising the dual-plane enhanced coil have identical wire arrangements. The relative positioning of the proposed coil with respect to the imaging target area, as well as the photograph of the proposed coil, is depicted in Figure 7.

## 3. Results and Discussion

### 3.1. Simulation Analysis

**Figure 8:**
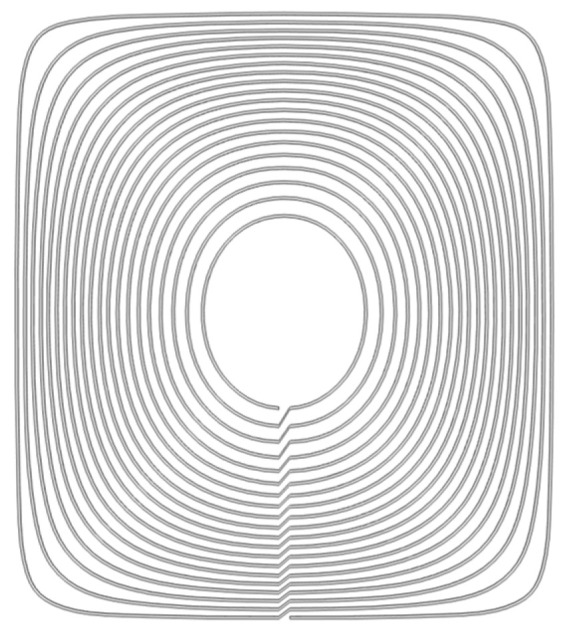
The simulation model of a single planar coil generated in Solidworks using the obtained conductor winding pattern.

The first step is to model the individual coils. The wire path layout obtained from the design is imported into Solidworks 2022 as a sketch reference, and the coil shape is drawn; the final coil model is constructed as shown in Figure 8.

**Figure 9:**
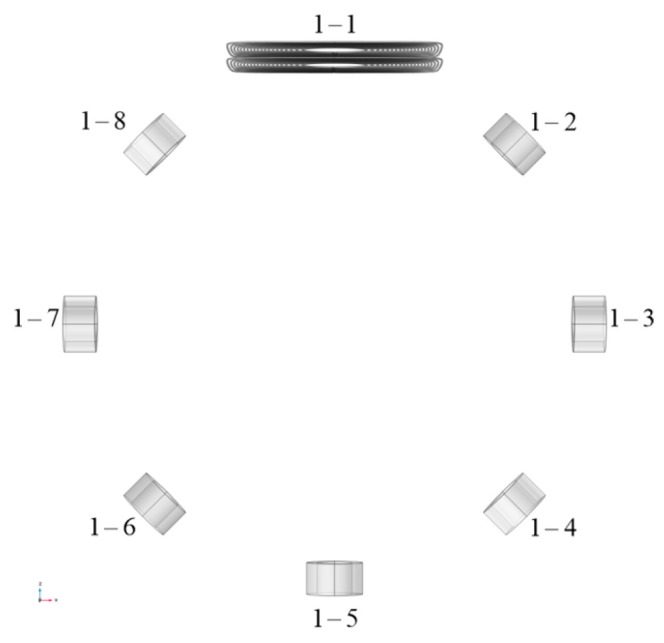
Detection model 1 constructed using the proposed coil.

The coil model is imported into COMSOL Multiphysics 6.0 and simulated and analyzed using the AC/DC module. First, the model of the dual-plane enhanced coil is constructed through mirroring operations, with a total of 40 turns. It is then used as the excitation coil, and the two planar coils are connected in series, with an excitation current of 1A. The detection coils consist of cylindrical coils with 100 turns each, totaling 7 coils evenly distributed around the imaging target area. The MIT detection model constructed using the proposed coils will be referred to as Model 1. Coil 1-1 is the proposed coil, and coils 1-2 to 1-8 as detection coils, as shown in Figure 9.

Simulations were conducted to compute the magnetic field strength within the imaging target area when stimulated by the proposed coil. This was conducted to verify if the computed magnetic field strength aligns with the values obtained through Equation (10), ensuring that the coils meet the design requirements. Next, a spherical disturbance object was placed inside it. When perturbation objects with different conductivities were positioned at various locations within the imaging target area, simulations were performed to analyze the induced voltage and phase signals received by coils 1-2 to 1-8 in model 1. The selected disturbance objects had conductivities of 0.82 S/m and 0.1762 S/m, representing abnormal tissues associated with cerebral hemorrhage and cerebral edema, respectively. This was carried out to assess the effectiveness of the proposed coil in detecting low-conductivity objects. The specific simulation parameters are shown in Table 1.

**Figure 10:**
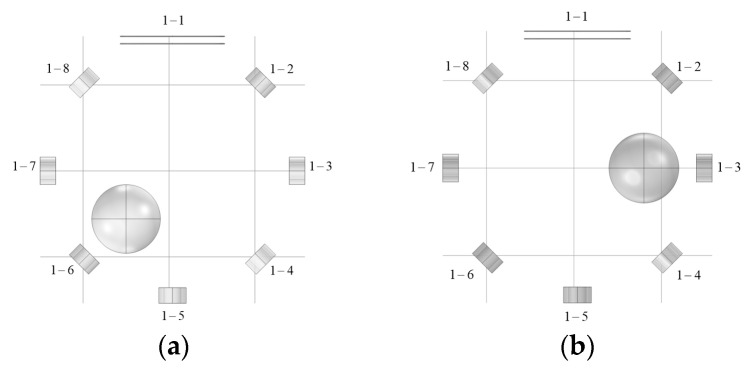
The location of the disturbance objects in Model 1 (unit: mm): (**a**) The disturbance object is located at (−28, 0, −75); (**b**) The disturbance object is located at (0, 0, 140).

Figure 10 illustrates the location of the disturbance object in Model 1.

### 3.2. Analysis of Results

**Figure 11:**
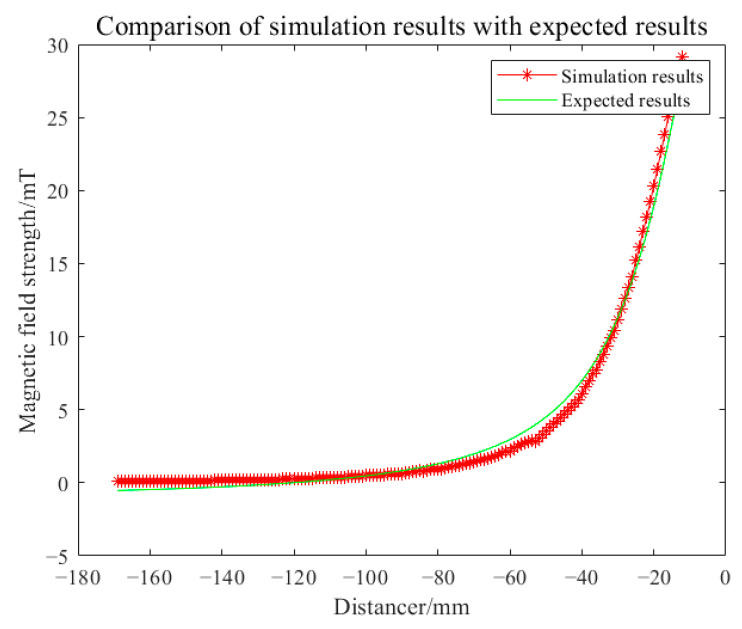
Comparison of simulated and theoretically calculated magnetic field intensity results for the proposed coil.

The simulation analysis of the proposed coil was carried out according to the simulation analysis steps in the previous subsection. First, the magnetic field strength generated within the imaging target area by the proposed coil was simulated at an excitation frequency of 1 MHz and excitation current of 1A. The simulated magnetic field strength was compared with the values calculated using Equation (10). The root mean square error between the two was found to be 0.01. The comparison results of magnetic field strength are illustrated in Figure 11.

**Figure 12:**
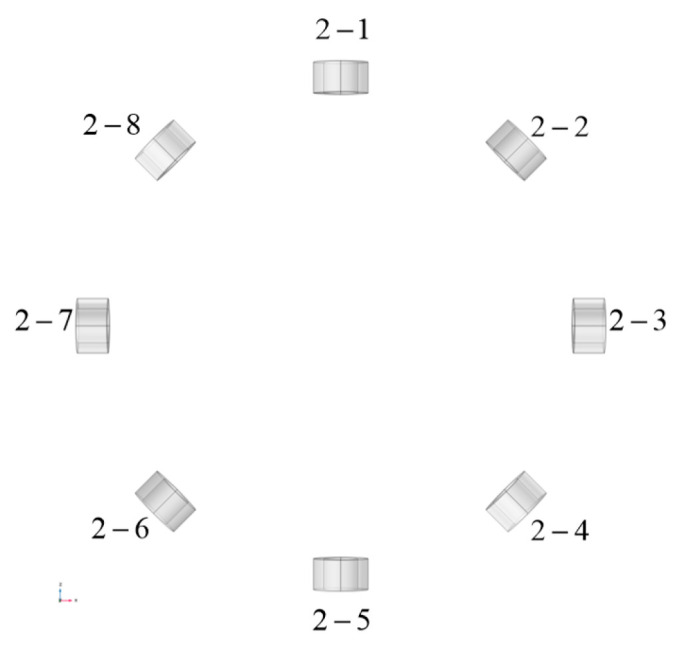
Detection model 2 constructed using cylindrical coils.

The main reasons for the errors are primarily attributed to the manual coil drawing in Solidworks, following the wire routing path shown in Figure 6, which introduces inherent inaccuracies. Additionally, when the coil model is imported into COMSOL, minor deformations occur. These deformations can lead to slight perturbations in the magnetic field distribution generated by the coil in space. The excitation coil in Model 1 was replaced with a cylindrical coil with 40 turns while keeping the detection coils unchanged, resulting in Model 2. This was carried out to demonstrate that the proposed coil is more suitable as an excitation coil for cerebral hemorrhage MIT compared to a cylindrical coil, as shown in Figure 12.

To compare the linearity of the magnetic fields produced by the two types of coils, we sampled points on the xz plane where x is 0 in both Model 1 and Model 2, along with their corresponding magnetic field strength values. The linear fit goodness was calculated separately for each case. The linearity of the magnetic field strengths produced by both was compared by evaluating the coefficient of determination $R^{2}$, defined as follows:

(15) $$R^{2}=1-\frac{\sum_{i=1}^{n}{\left(B_{i}-f_{i}\right)}^{2}}{\sum_{i=1}^{n}{\left(B_{i}-\bar{B}\right)}^{2}}$$

where n represents the sample size, $B_{i}$ denotes the magnetic field strength at different positions, $f_{i}$ represents the corresponding values obtained by fitting a linear function using $B_{i}$, and $\bar{B}$ is the mean of $B_{i}$. The final computation results are presented in Table 2.

The results indicate that the linearity of the magnetic field generated by the proposed coil in the imaging target area is 60.07% higher than that generated by the cylindrical coil. This enhancement effectively enhances the stability of the magnetic field and reduces field fluctuations and drift. It aids in acquiring more accurate information from the imaging target area to improve imaging quality. 

**Figure 13:**
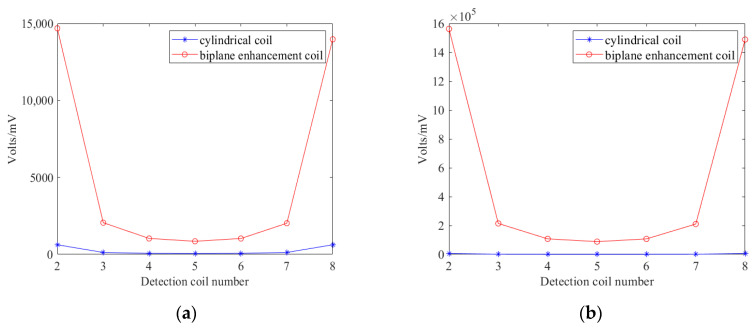
Comparison of detected voltage values of two types of coil in empty field: (**a**) When the excitation frequency is 1 MHz; (**b**) When the excitation frequency is 10 MHz.

Simulation analyses were conducted using the simulation parameters listed in Table 1. Initially, the detected voltage values received by the detection coil were compared under the condition that the simulation parameters such as excitation frequency and number of turns were identical for both Model 1 and Model 2, as shown in Figure 13.

Figure 13a and Figure 13b, respectively, illustrate the detected voltage values by the detection coil when the proposed coil and the cylindrical coil are used as the excitation coils at excitation frequencies of 1 MHz and 10 MHz, with no interfering objects present. As coil 5 is the farthest from the excitation source, it typically receives the weakest detection signals. The induced voltage values detected by coil 1-5 are two orders of magnitude higher than those detected by coil 2-5, effectively enhancing the detection signal strength and preventing the effective signal from being submerged in noise.

**Figure 14:**
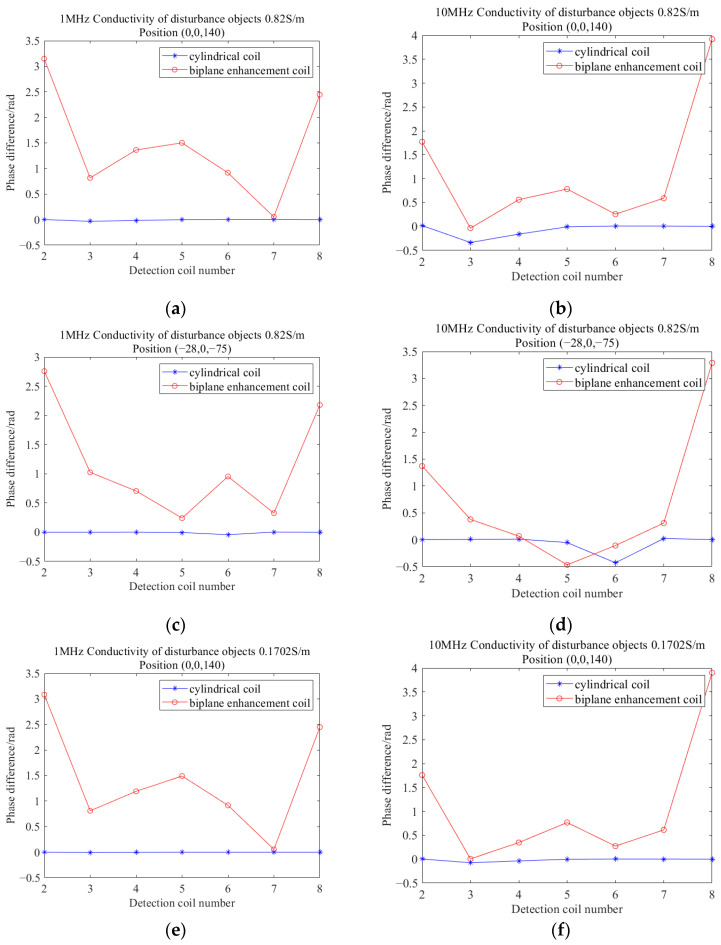

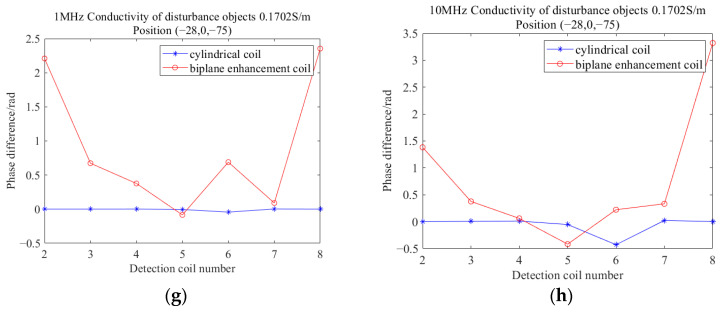
(**a**–**h**) are the comparison of the phase difference values of the detection coils corresponding to the two types of coils at different locations of the disturbing object with excitation frequencies of 1 MHz and 10 MHz, respectively. The conductivities of the interfering objects are 0.82 S/m and 0.1762 S/m, respectively.

Given that phase difference is commonly used for imaging in later stages, a comparison was made between the proposed and cylindrical coils when used as excitation coils, regarding the detected phase difference values. The directly calculated phase difference data often suffer from issues such as low numerical values and unclear trends, especially during the detection of low-conductivity objects. This makes it difficult to accurately reflect information such as the conductivity value, position, and shape of the measured object. In order to better reflect the distribution of conductivity in the target imaging area and analyze the characteristics of the object, it is necessary to normalize the phase difference data.

(16) $$\varphi_{s}=\frac{\varphi_{0}-\varphi_{d}}{\varphi_{0}}\cdot k$$

where $\varphi_{0}$ and $\varphi_{d}$ denote the phase information measured in the empty field and in the presence of a perturber, respectively, and are the gains, which usually take the value of 1000 [25]. Figure 14 illustrates the comparison of normalized phase differences measured in the presence of disturbance objects.

When the interfering object is located at (−28, 0, −75), it is close to coils 5 and 6, which corresponds to the larger phase fluctuations observed in coils 5 and 6 in the figure. When the interfering object is positioned at (0, 0, −140), it is close to coils 3 and 4, resulting in significant phase fluctuations observed in these coils in the figure. From the above figure, it can be observed that the phase difference values obtained from the detection model constructed using the proposed coil exhibit higher intensity. This enables a more effective reflection of the position information of the object, indicating higher sensitivity.

**Figure 15:**
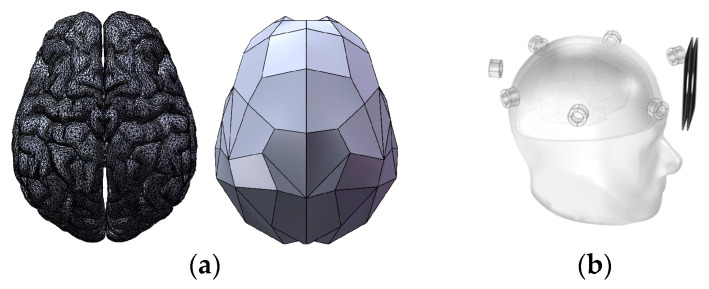
Constructed brain parenchyma model and human brain simulation detection model: (**a**) Real and simplified brain parenchyma models; (**b**) Four-layer human brain simulation test model.

Additionally, a four-layer brain tissue simulation model containing scalp, skull, cerebrospinal fluid, and brain parenchyma, as depicted in Figure 15, was established. This model was used to evaluate the effectiveness of the proposed coil in detecting abnormal low-conductivity tissues in the human body under conditions closer to reality. The geometry of the parenchymal part of the brain in the model is simplified, while preserving its overall characteristics. This is significant in cerebral hemorrhage MIT research. It allows for a more accurate simulation of the propagation and attenuation of magnetic fields in brain tissues, thereby further improving the accuracy and reliability of the results.

**Figure 16:**
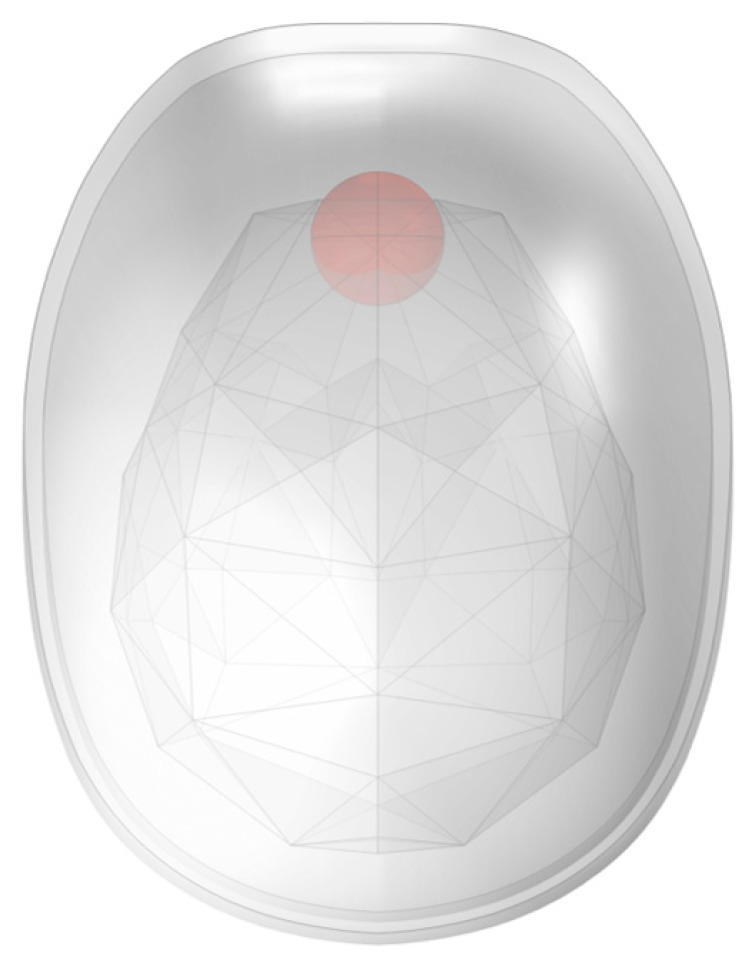
Schematic diagram of simulated bleed location.

A spherical region with a radius of 10 mm was chosen as the simulated hemorrhage location. It was located at the interface between the frontal side of the brain tissue and the cerebrospinal fluid. The simulated hemorrhage volume was approximately 4.19 mL, as shown in Figure 16.

The excitation frequency of 1MHz was selected for its ability to provide adequate penetration depth, particularly suitable for detecting large-scale biological tissues. Additionally, it avoids inducing significant thermal effects or other harmful impacts on the tissues, ensuring better patient safety. It is important for clinical applications such as dynamic monitoring. The constructed brain tissue simulation model and the conductivity of each part of the hemorrhage region are shown in Table 3.

During the detection process, the entire coil array was rotated successively at positions 1-1, 1-2, up to 1-7 to collect detection data. A total of 56 sets of measurements were obtained.

**Figure 17:**
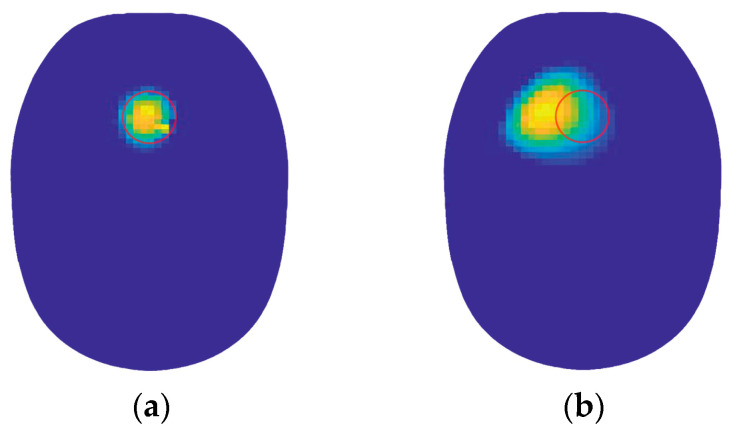
Reconstructed image of brain hemorrhage obtained when using the biplane enhancement coil and cylindrical coil for excitation: (**a**) Reconstructed image obtained from the excitation of the proposed coil; (**b**) Reconstructed image obtained from the excitation of the cylindrical coil.

The NR (Newton–Raphson) algorithm was utilized for image reconstruction, and the results are shown in Figure 17. The red circle represents the bleed region set by the simulation, with (a) and (b) displaying the reconstructed images of conductivity distributions obtained from the detected data of models 1 and 2, respectively.

From Figure 17, it can be observed that the hemorrhage region in the reconstructed image (a) closely matches the simulated hemorrhage region, with minimal deviation. Conversely, in the reconstructed image (b) obtained using detection data from the cylindrical coil excitation, numerous artifacts are present, and the position of the hemorrhage region exhibits deviation. The cause of this situation lies in the relatively weak magnetic field generated by the 40-turn cylindrical coil, resulting in poor linearity. As a result, the effective information in the detection signal is susceptible to noise interference. Additionally, the weak detection signal received further contributes to the significant error in the final imaging result. The effectiveness of image reconstruction using the proposed coil and cylindrical coil as excitation sources was evaluated by computing the correlation coefficient (ρ) between the actual conductivity distribution and the reconstructed images. The correlation coefficients for the reconstructed images obtained using both coils are presented in Table 4.

From Table 4, it is evident that the proposed coil exhibits superior detection performance, with a correlation coefficient improvement of 18.26% in the reconstructed images. This indicates a more accurate detection of the conductivity distribution within the imaging target area. The detection performance surpasses that of cylindrical coils with same parameters.

## 4. Conclusions

In this paper, a biplane enhancement coil that can be used for brain hemorrhage MIT technique is proposed by using the target field method, combined with the spatial magnetic field attenuation law set. Precise control of the magnetic field distribution in the imaging target region is realized. Through simulation analysis, it has been demonstrated that compared to cylindrical coils, this coil effectively enhances the linearity of the magnetic field within the imaging target area and increases the detection signal strength of the detection coils in MIT. Under the same simulation parameters, the linearity of the generated magnetic field has been improved by 60.07%, leading to an increase in the detected voltage magnitude by approximately two orders of magnitude. This provides more reliable data support for subsequent imaging. Additionally, a more realistic four-layer simulation model of the human brain is established to validate the effectiveness of the proposed coil for detecting cerebral hemorrhage regions. Under the simulated condition of a hemorrhage volume of 4.19 mL, imaging with the proposed coils yields superior results compared to cylindrical coils. The imaging artifacts are significantly reduced, leading to more accurate localization of the hemorrhagic region. The correlation coefficient of the reconstructed images improves by 18.26%, approaching the simulated hemorrhage location more closely. It helps to improve the imaging quality and provide more accurate imaging results. It can offer a more reliable and effective solution for detecting and locating low-conductivity targets. Subsequently, all detection coils will be replaced with the double-plane enhanced coils for simulation analysis and experimental setup to further demonstrate the effectiveness of the proposed coils in brain hemorrhage MIT detection imaging.

## Figures and Tables

**Table 1:** Simulation parameter settings.

Radius of Imaging Target Area (mm)	Excitation Current (A)	Excitation Frequency	Conductivity of Disturbance Objects (S/m)	Position of Disturbance Object (mm)
85	1	1	0.1762	(0, 0, 140) (−28, 0, −75)
			0.82	(0, 0, 140) (−28, 0, −75)
		10	0.1762	(0, 0, 140) (−28, 0, −75)
			0.82	(0, 0, 140) (−28, 0, −75)

**Table 2:** Comparison of the linearity of the magnetic field generated by the two types of coil.

Coil Type	$R^{2}$
biplane enhancement coil	0.738365
cylindrical coil	0.461274

**Table 3:** Conductivity of each part of the brain tissue simulation model.

Area	Scalp	Skull	Cerebrospinal Fluid	Brain Tissue	Hemorrhage Area
Conductivity (S/m)	0.044	0.024	2	0.0275	0.822
Relative permittivity	50.8	145	109	480	3030

**Table 4:** Image correlation coefficients of reconstructed images obtained using different coils for excitation.

Reconstructed Image	ρ
Reconstructed image(a)	0.9771
Reconstructed image(b)	0.8262

## Data Availability

Dataset available on request from the authors. The raw data supporting the conclusions of this article will be made available by the authors on request.
